# Automatic frequency and phase alignment of in vivo J-difference-edited MR spectra by frequency domain correlation

**DOI:** 10.1007/s10334-017-0627-y

**Published:** 2017-06-01

**Authors:** Evita C. Wiegers, Bart W. J. Philips, Arend Heerschap, Marinette van der Graaf

**Affiliations:** 1 Department of Radiology and Nuclear Medicine, Radboud university medical center, 767, PO Box 9101, 6500 HB Nijmegen, The Netherlands; 2 Department of Pediatrics, Radboud university medical center, Nijmegen, The Netherlands

**Keywords:** J-difference editing, Spectral alignment, Lactate, Magnetic resonance spectroscopy

## Abstract

**Objective:**

J-difference editing is often used to select resonances of compounds with coupled spins in ^1^H-MR spectra. Accurate phase and frequency alignment prior to subtracting J-difference-edited MR spectra is important to avoid artefactual contributions to the edited resonance.

**Materials and methods:**

*In*-*vivo* J-difference-edited MR spectra were aligned by maximizing the normalized scalar product between two spectra (i.e., the correlation over a spectral region). The performance of our correlation method was compared with alignment by spectral registration and by alignment of the highest point in two spectra. The correlation method was tested at different SNR levels and for a broad range of phase and frequency shifts.

**Results:**

In-vivo application of the proposed correlation method showed reduced subtraction errors and increased fit reliability in difference spectra as compared with conventional peak alignment. The correlation method and the spectral registration method generally performed equally well. However, better alignment using the correlation method was obtained for spectra with a low SNR (down to ~2) and for relatively large frequency shifts.

**Conclusion:**

Our correlation method for simultaneously phase and frequency alignment is able to correct both small and large phase and frequency drifts and also performs well at low SNR levels.

## Introduction

Because of a limited chemical shift range and a high number of proton-containing metabolites, a typical in vivo ^1^H-MR spectrum of the brain contains many overlapping peaks. This complicates the assignment and quantification of specific metabolite signals that (partly) co-resonate with signals of other metabolites. Selection of resonances by J-difference editing is widely used to overcome this problem for J-coupled spin systems [1–3]. J-difference editing involves the consecutive acquisition of two spectra in which frequency-selective radiofrequency (RF) pulses are alternatively switched on and off on one resonance of the coupled spin-system. For doublet resonances the echo time (TE) is set to the reciprocal of the J-coupling constant of the spins of interest (TE = 1/J), and for triplet resonances to TE = 1/2 J [3]. Due to the frequency-selective RF pulse, the evolution of the J-coupling of the spin system is refocused, whereas the J-coupling evolves throughout the echo time when the selective RF pulses are switched off or placed off-resonance. The so-called ‘on’ and ‘off’ spectra are subtracted to eliminate all unaffected resonances, leaving only those of the selectively refocused coupled spins for quantification.

Small phase and/or frequency differences between the ‘on’ and ‘off’ spectra will occur due to system instabilities between the two acquisitions (e.g., by *B*_0_ drift or subject movement) or caused by the selective inversion RF pulses. If uncorrected, these shifts result in artifacts and subtraction-errors in the difference spectra that may affect the appearance and, thus, the quantification of the edited resonances. Correction is commonly performed by alignment using a large peak in the spectrum as a reference before averaging or subtracting (e.g., in [4, 5]), or by consecutive phase alignment by a least-squares algorithm and frequency alignment by a cross-correlation algorithm (e.g., in [6, 7]). Furthermore, the ‘on’ and ‘off’ spectra are often separately aligned (e.g., in [8, 9]), which may leave residual intensity in the difference spectra and, therefore, hampers quantification of the edited peaks. Recently, Near et al. presented an alignment method, referred to as ‘spectral registration’, in which frequency and phase drifts in MRS data were corrected simultaneously in the time domain [10]. Their algorithm minimizes the difference between the first part of a free induction decay (FID) with varying phase and frequency terms, and a reference FID.

We propose a new correction method for simultaneous phase and frequency alignment in the frequency domain, which is applicable to a complete dataset comprising both the ‘on’ and ‘off’ spectra. Our alignment method makes use of the scalar product of spectra (i.e., the correlation over a spectral region) to express how closely they are aligned. It aims for alignment of a relative large spectral region, excluding the part where the frequency selective RF pulses are placed and excluding the water signal and signal free regions. The performance of the method is tested at different SNR levels and for a broad range of phase and frequency shifts. The method is demonstrated for the in vivo J-difference editing of the methyl resonance of lactate and the methylene resonance of γ-aminobutyric acid (GABA) at 3.0 ppm in the human brain.

## Materials and methods

The proposed correlation method was developed as part of a study in which brain lactate levels were determined [11]. As such, the method has been extensively tested on J-difference-edited spectra of brain lactate. The evaluation and noise simulation of the alignment procedures of spectra were performed in Matlab (Matlab 2016a, The MathWorks Inc.).

### Alignment by the correlation method

In the correlation method the first recorded spectrum in a series was chosen as the reference spectrum (*S*_ref_), to which all other spectra of the dataset (*S*_*n*_) were aligned. The scalar product of *S*_ref_ with *S*_*n*_ was calculated with varying phase shifts ($\Delta \varphi$) and simultaneously varying frequency shifts ($\Delta f$) of *S*_*n*_, where the scalar product of two complex vectors $a = \left[ {a_{1} \ldots a_{n} } \right]$ and $b = \left[ {b_{1} \ldots b_{n} } \right]$ was defined as $a \cdot b = \mathop \sum \nolimits_{i = 1}^{n} \bar{a}_{i} b_{i}$. Only the spectral region marginally affected by the frequency-selective inversion pulses was used in the alignment algorithm. The optimal phase and frequency shifts was be found by maximizing the normalized scalar product [C(∆φ, ∆*f*)] between both spectra (Eq. 1).

1 $$C\left( {\Delta \varphi ,\Delta f} \right) = \frac{{{\text{Re}}\left( {S_{n} \left( {f + \Delta f} \right)e^{ - i\Delta \varphi } \Delta S_{\text{ref}} } \right)}} {{\left\| {{S_{\text{ref}}}} \right\|} \; {\left\| {S_{n} \left( {f + \Delta f} \right)e^{ - i\Delta \varphi }} \right\|}}$$

### MRS protocol for J-difference editing of lactate

The study involving lactate editing included 13 healthy volunteers (six males, age 26.2 ± 5.5 years) and informed consent was obtained from all participants. MRS measurements were performed at 3.0 T (Tim MAGNETOM Trio; Siemens, Erlangen, Germany), using a 12-channel head coil for signal reception. A T_1_-weighted anatomical image (MPRAGE; 256 × 256 mm^2^ field of view, 256 slices, 1 mm^3^ voxels) was acquired for voxel localization.

Brain lactate was measured from a 22.5–25.0 cm^3^ voxel located in the periventricular and supraventricular region with an interleaved J-difference editing [12] semi-LASER spectroscopy sequence [13] with an echo time (TE) of 144 ms, a repetition time (TR) of 3000 ms, an acquisition time (TA) of 1:42 min (two dummy scans and 32 averages) and a bandwidth of 1200 Hz (spectral resolution: 1200 Hz/1024 = 1.17 Hz). J-difference editing was performed with frequency selective inversion pulses (MEGA) with a bandwidth of 75 Hz and a duration of 30 ms, centered on the lactate quartet at 4.1 ppm (resulting in the ‘on spectrum’). In the second acquisition, the frequency selective inversion pulses were centered at −1.5 ppm (resulting in the ‘off spectrum’). ‘On’ and ‘off’ spectra were acquired in blocks of 32 averages. Three to five ‘on’ and ‘off’ spectra were acquired per subject, resulting in a total number of 59 ‘on’ and ‘off’ spectra.

All spectra were first zero-filled from 1024 to 2048 data points and a Fourier transform was applied. Subsequently, spectra were aligned with the method described above. The performance of our correlation method was compared with alignment by spectral registration (part of the open source MATLAB based software toolkit FID-A [10, 14]), which was applied over the same frequency range (i.e., from 1.6 to 3.4 ppm), and by frequency alignment of the highest point in *S*_*n*_ with the highest point in *S*_ref_. The latter is equivalent to the automatic alignment function in jMRUI [15]. Also, difference spectra were obtained without prior alignment. All difference spectra were apodized with a 5 Hz Lorentzian.

As an estimate of the quality of the alignment, the integral of the absolute intensity of the difference spectrum between 1.6 and 3.4 ppm (*ɛ*_alignment_) was calculated per difference spectrum for each alignment strategy.

The three or five difference spectra per subject were averaged to increase the signal-to-noise ratio for quantification. The lactate doublet in the averaged difference spectra were fitted with the AMARES algorithm in jMRUI. As a measure of the precision of the fit of the lactate doublet, the coefficient of variation (CoV) of the fit was calculated by dividing the standard deviation of the amplitude by the amplitude. Fits with a CoV >30% were discarded.

To assess differences between alignment strategies, ANOVA followed by Bonferroni post hoc tests were performed on the CoV’s. A *p* value less than 0.05 was considered statistically significant.

### MRS protocol for the J-difference editing of GABA

In addition, we applied the correlation method to J-difference-edited spectra of GABA optimized for detection of the GABA signal at 3.0 ppm. Spectra were acquired form a healthy volunteer from an 8 cm^3^ voxel with a MEGA-PRESS sequence (TR = 1500 ms; TE = 68 ms; 128 averages; spectral width = 1200 Hz) at 3.0 T (Tim MAGNETOM Trio; Siemens, Erlangen, Germany). The frequency-selective refocusing pulses, with a bandwidth of 50 Hz, were placed at 1.9 ppm (‘on’ spectrum) and subsequently at 7.5 ppm (‘off’ spectrum). Spectra were zero-filled from 512 to 1024 data points. ‘On’ and ‘off’ spectra were aligned using the correlation method over the spectral region from 2.8 to 4.1 ppm. Furthermore, to evaluate the performance of the correlation method on spectra with both upright and inverted peaks, alignment was performed on two GABA difference spectra. These spectra were acquired by subtracting the ‘on’ and ‘off’ spectra using manufacturer-specific software. A frequency shift of 6 Hz and a phase shift of 15° was applied to one of the spectra (*S*_*n*_), prior to alignment. Alignment of these spectra with the correlation method was performed over the spectral region from 0.5 to 4.2 ppm.

### Effect of noise

One ‘off’ spectrum from the J-difference-edited lactate spectra was used to evaluate the performance of the proposed correlation method at different SNR levels. This spectrum (*S*_ref_) was zero-filled to 4096 points and duplicated to create a spectrum, which has to be aligned (*S*_*n*_). Normally distributed random noise was added to *S*_ref_ and *S*_*n*_ separately to generate spectra with an SNR of 30, 10, 3, 2 and 1.5. SNR was defined as the maximum of the amplitude of the NAA-peak divided by the standard deviation of the noise. A frequency drift (applied in the time domain, from −30 to 30 Hz in 2 Hz steps) and a phase drift (−20° to 20° in 2.5° steps) was applied on each average of *S*_*n*_. For each SNR value this procedure was repeated 25 times. Frequency and phase drift correction was performed on *S*_*n*_ using the correlation method as well as spectral registration. Only the spectral region from 1.6 to 3.4 ppm was used in both alignment methods. The performance of the two alignment methods was evaluated by calculating the phase estimation error and frequency estimation error, defined as the absolute difference between the true phase or frequency drift and estimated phase or frequency drift.

## Results

For all J-difference-edited lactate spectra, a clear maximum of the normalized scalar product was found for a certain phase and frequency shift. The mean (±SD) normalized scalar product between *S*_ref_ and *S*_*n*_ after aligning the spectra with our alignment method (correlation method) was 0.95 ± 0.08. The SNR of an in vivo spectrum of 32 averages was approximately 90.

**Fig. 1:**
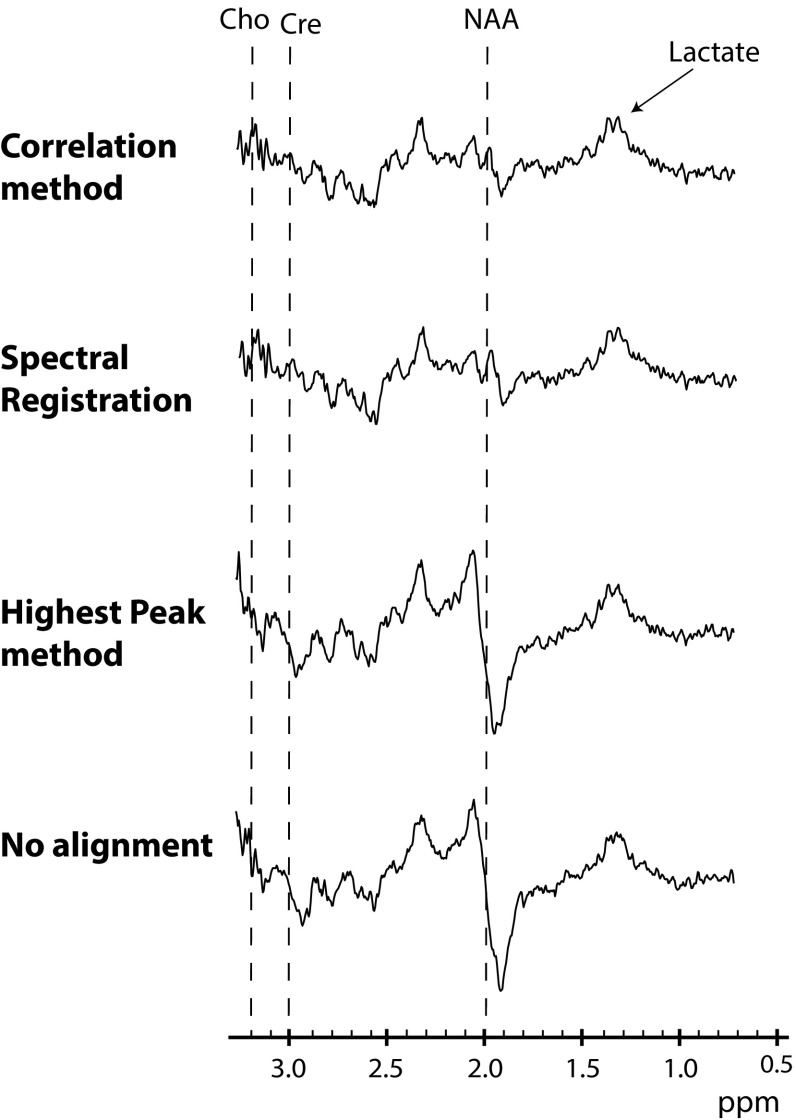
Averaged difference spectra. An example of difference spectra, obtained without aligning the ‘on’ and ‘off’ J-difference-edited lactate spectra before subtraction (*bottom*), by aligning the highest peak in both spectra (*third row*), by aligning with spectral registration (*second row*) and by our correlation method, i.e., maximizing the normalized scalar product between the ‘on’ and ‘off’ spectra (*top row*). The lactate doublet in the final difference spectra at 1.3 ppm is indicated. Subtraction errors are present around the choline (3.2 ppm; Cho), creatine (3.0 ppm; Cre) and NAA (2.0 ppm) frequencies (indicated with *dashed lines*) when no alignment protocol or the highest peak method is used, but are greatly reduced after alignment with spectral registration and after aligning with our correlation method

Typical difference spectra of one volunteer, depicting the lactate doublet at 1.3 ppm, obtained without alignment, alignment with the highest peak (HP) method, alignment using spectral registration and alignment with the correlation method are shown in Fig. 1. Without prior alignment or when spectra were aligned with the HP method, large subtraction errors are present, especially at the spectral regions around NAA (2.0 ppm), creatine (Cre; 3.0 ppm) and choline (Cho; 3.2 ppm). These subtraction errors are greatly reduced when spectral registration or the correlation method was used for aligning the ‘on’ and ‘off’ spectra, as confirmed by a lower ɛ_alignment_ (no alignment: 76.0 ± 38.8; HP method: 67.9 ± 36.9; spectral registration: 38.2 ± 6.4; correlation method: 39.2 ± 6.6).

**Fig. 2:**
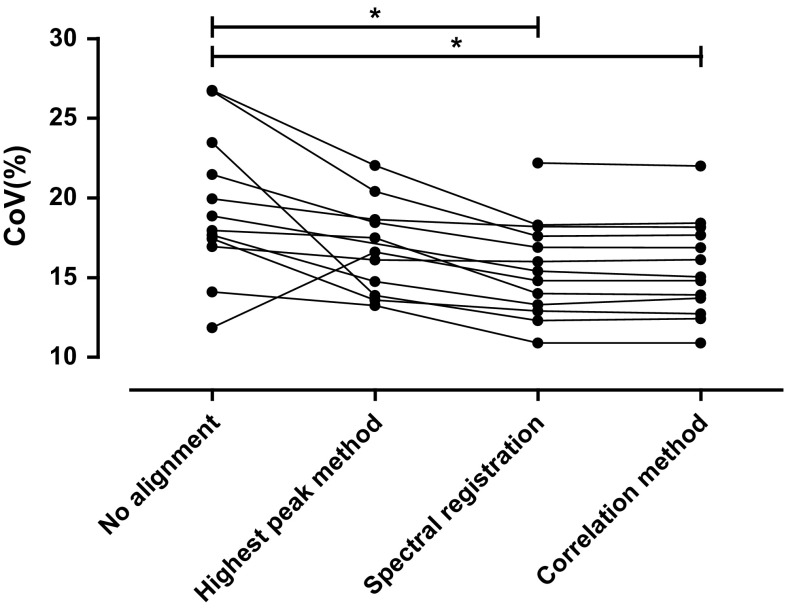
Assessment of alignment quality. The coefficient of variation (CoV) of the fit of the lactate doublet at 1.3 ppm was calculated per averaged difference spectrum for each alignment strategy. No alignment, highest peak method, spectral registration and the correlation method are compared. *p < 0.05

The lactate doublet at 1.3 ppm in the final averaged difference spectra was fitted reliably in all difference spectra acquired with spectral registration and with the correlation method (i.e., CoV <30%), whereas the lactate doublet could not be fitted reliably in one difference spectrum acquired without prior alignment and in two difference spectra aligned with the HP method. The precision of the lactate doublet fit increased after aligning the spectra using spectral registration and by using the correlation method, as shown by a decreased CoV of the fit (mean CoV ± SD: 19.43 ± 4.5, 16.84 ± 2.8, 15.60 ± 3.0 and 15.60 ± 3.02% for no alignment, HP method, spectral registration and correlation method respectively; *p* < 0.05 correlation method vs. no alignment and spectral registration vs. no alignment; Fig. 2).

**Fig. 3:**
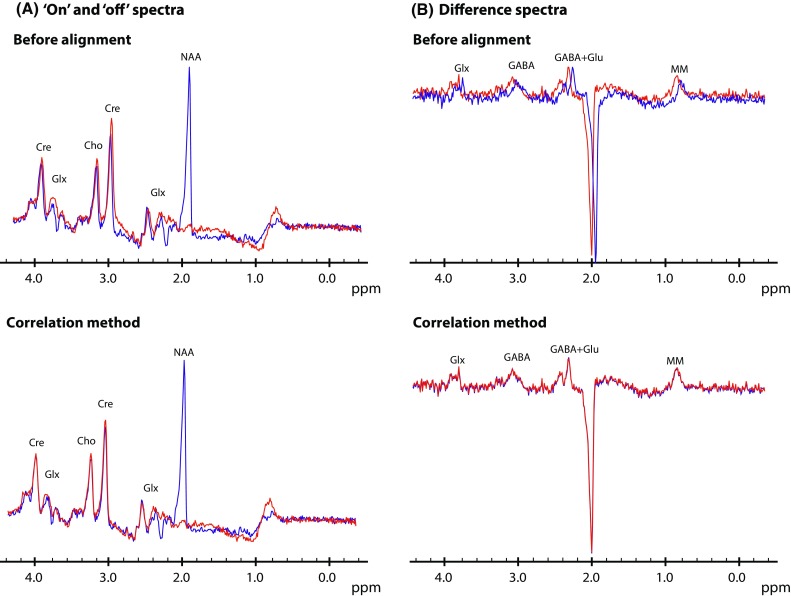
Alignment of J-difference-edited GABA spectra before and after subtraction. **a** GABA ‘on’ (*red*) and ‘off’ (*blue*) spectrum before alignment (*top row*) and after alignment with the correlation method (*bottom row*). **b** *Top row* two typical GABA difference spectra before alignment (*S*_ref_ in *red* and *S*_*n*_ in *blue*). A frequency shift of 6 Hz and a phase shift of 15 degrees was applied to *S*_n_. *Bottom row S*_ref_ and *S*_*n*_ after alignment with the correlation method. *Cre* creatine, *Glx* glutamate and glutamine, *Cho* choline, *NAA N*-acetylaspartate, *GABA* γ-aminobutyric acid, *MM* macromolecular resonances

The performance of the correlation method was tested on in vivo J-difference-edited GABA spectra (Fig. 3a). Visual inspections show that alignment of the ‘on’ and ‘off’ spectra works properly. Also, alignment of GABA-edited difference spectra (Fig. 3b), comprising both inverted and upright peaks, is successful using the correlation method.

**Fig. 4:**
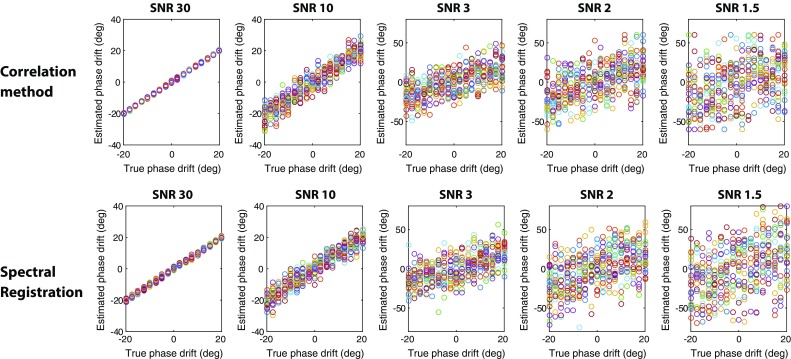
Phase drift estimation. Actual and estimated phase drift, obtained by the correlation method (*top row*) and by spectral registration (*bottom row*), at five different SNR levels (SNR of 30, 10, 3, 2 and 1.5) over 25 repetitions

**Fig. 5:**
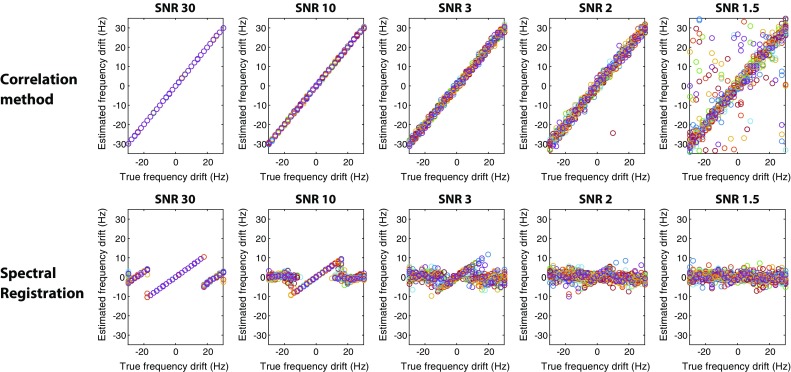
Frequency drift estimation. Actual and estimated frequency drift, obtained by the correlation method (*top row*) and by spectral registration (*bottom row*), at five different SNR levels (SNR of 30, 10, 3, 2 and 1.5) over 25 repetitions

**Fig. 6:**
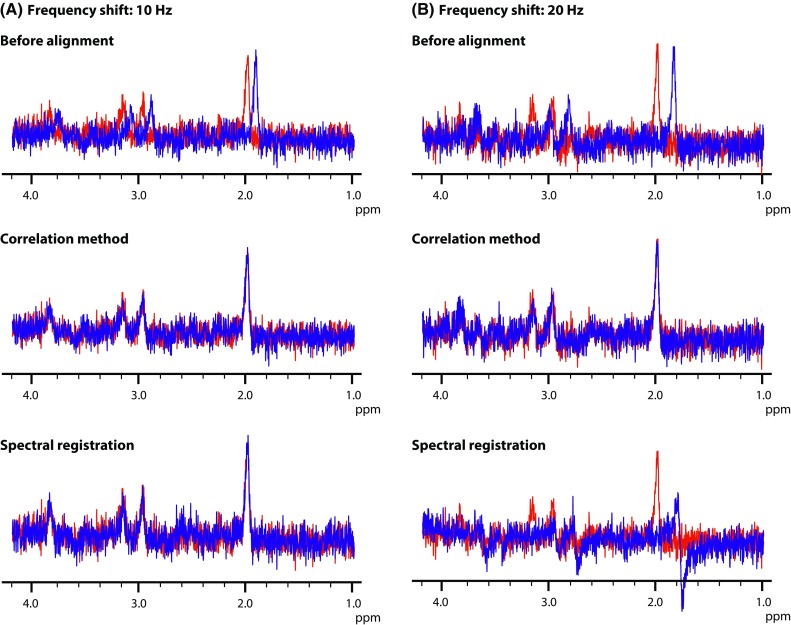
Alignment of spectra with a frequency shift of 10 Hz and 20 Hz. *Top row* example of two spectra (*S*_ref_ in *red* and *S*_*n*_ in *blue*) with an SNR of 10 before alignment. A frequency shift of 10 Hz (**a**) and 20 Hz (**b**) was applied on *S*_*n*_. *Middle row S*_ref_ (*red*) and *S*_*n*_ (*blue*) after alignment using the correlation method. Spectra are accurately aligned for both frequency shifts. *Bottom row S*_ref_ and *S*_*n*_ after alignment using spectral registration. Alignment is successful for a frequency shift of 10 Hz. For a frequency shift of 20 Hz, spectral registration erroneously aligns the choline peak in *S*_*n*_ with the creatine peak at 3.0 ppm in *S*_ref_

Upon comparison of the performance of the correlation method and spectral registration for alignment of spectra with increasing noise, the phase estimation errors were comparable for both methods (Fig. 4; Table 1). For both methods the phase estimation error increases with decreasing SNR and phase estimation appears to be not accurate for an SNR below 3. The correlation method can accurately estimate the frequency drift throughout the whole frequency drift range, which is not the case for the spectral registration method (Fig. 5). For larger frequency shift (e.g. >20 Hz for an SNR of 10) the spectral registration technique does not align the correct peaks (Fig. 6). As such, the frequency estimation error was calculated for both methods only over the frequency range in which spectral registration worked properly (Table 2). In this range, the frequency estimation error is comparable for both alignment methods until an SNR of 3. For an SNR below 3, the frequency estimation error is smaller for the correlation method compared with spectral registration.

**Table 1:** Phase estimation error (degree)

	SNR 30	SNR 10	SNR 3	SNR 2	SNR 1.5
Correlation method	0.26 ± 0.20	3.39 ± 2.66	11.21 ± 8.09	15.20 ± 11.54	21.25 ± 15.64
Spectral registration	0.48 ± 0.36	3.05 ± 2.30	11.17 ± 8.58	17.41 ± 12.78	24.48 ± 18.21

Absolute difference between the true phase drift and estimated phase drift. Data are mean ± SD

**Table 2:** Frequency estimation error (Hz)

	SNR 30	SNR 10	SNR 3	SNR 2	SNR 1.5
Correlation method	0.12 ± 0.0	0.25 ± 0.19	0.78 ± 0.62	1.29 ± 0.97	4.00 ± 7.41
Spectral registration	0.03 ± 0.02	0.29 ± 0.91	0.91 ± 0.79	2.15 ± 1.82	n/a

Absolute difference between the true frequency drift and estimated frequency drift, calculated over the frequency rang in which spectral registration works properly (see Fig. 5). Data are mean ± SD

## Discussion

Here we present a simple and robust method for simultaneous phase and frequency alignment of in vivo J-difference-edited MR spectra. Proper alignment reduces subtraction errors and increases the precisions of the fit of the metabolite signal of interest. This is especially of importance when the signal of interest is small, e.g., in the case of cerebral lactate, which is normally present at a tissue concentration of about 0.5 mM [16]. Also for reliable quantification of other compounds such as GABA, of which the resonances overlap significantly with large resonances of other metabolites, proper alignment of J-difference-edited spectra is important.

We compared our alignment method against a more conventional method in which the highest peaks in two spectra are aligned [15] and with spectral registration [10]. The limited spectral resolution of our dataset might have resulted in a slight underperformance of the highest peak method. The performance of the spectral registration method regarding subtraction errors and the precision of the fit of the lactate doublet, is comparable with the correlation method. However, our correlation method, appears to be more robust for correcting relatively large frequency drifts (i.e. >20 Hz). If such large frequency drifts are present, spectral registration may erroneously align, for example, the choline peak (at 3.2 ppm) with the creatine peak (at 3.0 ppm), as the frequency difference between these resonances is slightly more than 20 Hz at 3 T. This does not occur when using the correlation method. Furthermore, at lower SNR levels, frequency drifts (but not phase drifts) are corrected more accurately using the correlation method compared with spectral registration. Also, as described by Near et al., spectral registration may not be suitable for correcting a phase drift, if the first point of the FID of the signal is close to zero. This may be the case when the net intensity of the spectrum is close to zero due to the presence of both positive and negative peaks, as in, for example, GABA-edited difference spectra. We showed that the correlation method is capable of properly aligning such spectra.

Various other alignment strategies have been previously described, based on prior peak fitting or modeling of a singlet peak such as those from NAA [17] or Cre [4, 18], or based on principal component analysis (PCA) [5, 19]. The latter has been demonstrated to be very robust, but is usually also applied on a predefined single resonance peak. These methods are, therefore, not directly applicable when such a reference peak is not present, for example, in spectra obtained from tumors or in X-nuclei MR spectra. Our alignment algorithm is model-free and uses a broad spectral range. This makes our method very easy to be implemented, independent of distortions or of a poor SNR, and it does not require the presence of a distinct reference peak.

As the correlation method works in the frequency domain, the frequency estimation error is computationally limited by the vector size of the spectrum. As such, the minimal frequency estimation error which can be obtained is approximately equal to half of the spectral resolution. This is not the case for spectral registration, where the frequency estimation error can be (in theory) equal to zero. However, this limitation of the correlation method can be overcome by zero-filling. Another constraint is that the spectral region used for the grid search in the correlation method, should be only marginally affected by the frequency-selective RF pulses, to warrant spectral similarity. Model-based alignment methods may not suffer from this limitation. With regard to these two constraints, our proposed alignment method may also be valuable for aligning conventional non-edited MR spectra, e.g., for a time series prior to averaging.

## Conclusion

We demonstrated that maximizing the normalized scalar product between two spectra (i.e., the correlation over a spectral region) is a robust method for spectral alignment, an essential procedure prior to spectral subtraction in J-difference-edited MRS. The presented correlation method corrects both small and large phase and frequency drifts accurately and performs well at an SNR down to approximately 2.
